# Saint Louis University GWEP Pivots to Virtual Service Delivery: Lessons Learned

**DOI:** 10.1093/geroni/igab046.1831

**Published:** 2021-12-17

**Authors:** Marla Berg-Weger, Max Zubatsky, John Morley

**Affiliations:** 1 Saint Louis University, Saint Louis, Missouri, United States; 2 Medical Family Therapy Program, Saint Louis University, Missouri, United States; 3 Division of Geriatric Medicine, Saint Louis University School of Medicine, Missouri, United States

## Abstract

In response to the COVID-19 pandemic, Saint Louis University GWEP quickly pivoted service initiatives to online formats. Despite challenges of technology literacy and access, GWEP faculty, staff, and students creatively adapted in-person programming to online delivery and developed new virtually-delivered services. These service delivery adaptations provided opportunities for educating students, residents, faculty, community partners, and older adults and their caregivers to gain new knowledge and skills while continuing to participate in programming. This presentation will highlight innovations in the area of services to persons with dementia through Cognitive Stimulation Therapy, caregivers through education and support programs, older adults experiencing loneliness and social isolation through Circle of Friends, and older adults and caregivers through a virtual geriatric assessment clinic. We share highlights here of our efforts to pivot programming, access new funding streams, and, in some cases, create online delivery, including valuable lessons learned.

